# Knowledge, attitudes, and practices regarding enteral nutrition interruption management in intensive care units: a multicenter cross-sectional study predominantly among nurses

**DOI:** 10.3389/fnut.2026.1888995

**Published:** 2026-10-02

**Authors:** Quanquan He, Weiwei Ni, Maoyun Miao, Jiani Qian, Haolin Dou, Ningning Qin, Guosheng Han, Yulei Ma, Huizhen Dai

**Affiliations:** 1 Nursing Department, Weifang Key Laboratory for Clinical Nursing Research, Affiliated Hospital of Shandong Second Medical University, Weifang, China; 2 School of Nursing, Shandong Second Medical University, Weifang, China; 3 Intensive Care Unit, Affiliated Hospital of Shandong Second Medical University, Weifang, China; 4 Department of Emergency Medicine, Affiliated Hospital of Shandong Second Medical University, Weifang, China

**Keywords:** enteral nutrition, enteral nutrition interruption, healthcare professionals, intensive care unit, path analysis, training

## Abstract

**Background:**

Enteral nutrition (EN) is essential for critically ill patients, yet enteral nutrition interruption (ENI) is common in intensive care unit (ICU) and compromises nutrient delivery. ENI reflects clinical conditions, workflows, team collaboration, and bedside implementation, but how knowledge, attitudes, and practices (KAP) interact remains unclear.

**Objective:**

To assess ENI-related knowledge, attitudes, and practices among ICU healthcare professionals, predominantly nurses, and examine their interrelationships and associations with training, demographic, and organizational factors.

**Methods:**

This multicenter cross-sectional survey included 478 ICU healthcare professionals from 14 hospitals in Shandong and Henan, China, between December 2025 and February 2026; 93.5% were nurses. Self-reported KAP were assessed using a validated questionnaire. Descriptive statistics, Spearman correlation, multiple linear regression, and observed-variable path analysis were conducted.

**Results:**

Self-assessed knowledge scores were lower than attitude and practice scores, particularly for items related to the operational definitions of ENI. The three KAP domains were positively correlated, with the strongest correlation between knowledge and practice (ρ = 0.738, *p* < 0.001). Path analysis suggested that knowledge was associated with both attitudes and practices and that attitudes were also associated with practices. Bootstrap analysis further indicated a possible indirect association between knowledge and practice through attitudes (indirect effect = 0.339, 95% CI 0.246–0.438). EN-related training was associated with higher scores across all three domains, whereas EN team membership was associated with higher knowledge and practice scores. Several demographic and hospital characteristics showed domain-specific associations. The regression models explained a modest proportion of the observed variance (adjusted *R*^2^ = 0.054–0.141).

**Conclusion:**

Among ICU healthcare professionals, predominantly nurses, self-reported ENI management practices were associated with knowledge and attitudes, with attitudes potentially playing an intermediary role. EN-related training was also associated with more favorable KAP scores, while organizational and workflow factors may contribute to the observed variation. These findings may help identify potential targets for future intervention development and evaluation but do not establish causal relationships or improvements in actual ENI management, nutrient delivery, or patient outcomes.

## 1 Introduction

EN is the preferred route of nutritional support for critically ill patients and an essential component of comprehensive intensive care (1). Guidelines from the American Society for Parenteral and Enteral Nutrition (ASPEN) (1) and the European Society for Clinical Nutrition and Metabolism (ESPEN) (2) recommend that, in the absence of contraindications, EN should be initiated early and delivered in a standardized manner to provide necessary energy and nutrients, enhance immune function, modulate inflammatory responses, attenuate skeletal muscle catabolism, promote tissue repair, and maintain the integrity of the gastrointestinal and respiratory mucosal barriers, thereby improving clinical outcomes. However, in routine clinical practice, ICU patients often receive only approximately 60% of their prescribed nutritional targets, and ENI is one of the major barriers to achieving adequate energy and protein delivery (3).

ENI is defined as an interruption lasting at least one h during continuous EN infusion, or, in the context of intermittent feeding, failure to deliver the expected nutritional amount within 30 min when feeding is prescribed three times daily for 30 min each time (4, 5). In the ICU, EN is frequently interrupted because of diagnostic or therapeutic procedures, gastrointestinal intolerance, feeding-tube problems, hemodynamic instability, and healthcare-related factors, making it difficult to achieve target feeding in a timely manner (6). Previous studies have reported that the incidence of ENI ranges from 4.7% to 100%, with an average of approximately 48.3%; each patient experiences an average of 2–3 interruptions, with a cumulative interruption duration of approximately 33.8 h (6). A considerable proportion of these interruptions are related to avoidable workflow- and implementation-related factors (7, 8). These findings indicate that ENI is not driven solely by patients' clinical conditions but is also shaped by clinical workflow management, team collaboration, and bedside implementation (6, 9).

In this process, ICU healthcare professionals are the core implementers of EN delivery and interruption management (10–12). Nurses, who remain continuously at the bedside, play the most direct and sustained role in key steps such as EN initiation, suspension, and resumption (9, 10). They are responsible not only for EN screening, assessment, implementation, and monitoring, but also for timely resumption of feeding after diagnostic or therapeutic procedures and for managing gastrointestinal intolerance and feeding-tube-related problems (10). Therefore, although ENI management involves multidisciplinary collaboration, it largely manifests as a nurse-led bedside implementation process (10). Previous evidence suggests that failure to resume EN in a timely manner is an important contributor to ENI, and this process is highly dependent on the timeliness and standardization of nursing practice (9, 11). Accordingly, the KAP of ICU healthcare professionals, particularly nurses, are directly related to the quality and consistency of ENI management (9, 12).

Despite continuous updates to clinical guidelines, several important gaps remain in the existing literature (13, 14). First, previous studies have mainly focused on general EN management or related complications, with insufficient attention to ENI as a critical issue characterized by workflow dependence, contextual complexity, and team-based implementation (14–16). Second, existing evidence has largely remained descriptive, focusing on the current status of knowledge, attitudes, or practices, while offering limited explanation of how these domains are interrelated and associated with clinical practice (15, 17). Third, although training is widely regarded as relevant to clinical practice, its relationships with the KAP domains remain insufficiently understood, including whether it is associated with practice independently or through its associations with knowledge and attitudes (9, 12, 16). Therefore, beyond single-domain or descriptive investigations, the field of ENI management requires a more theory-informed approach to examine how these domains are associated with clinical practice and to inform intervention development.

Against this background, we constructed an analytical framework based on Knowledge Translation Theory and the Theory of Planned Behavior (18, 19). Knowledge Translation Theory emphasizes that clinical evidence can enter practice only after contextual adaptation, barrier identification, and transformation into implementation strategies (18, 20). The Theory of Planned Behavior further suggests that attitudes serve as a key bridge in the translation of knowledge into practice (19, 21). Integrating these two theories provides a framework for examining how knowledge, attitudes, contextual conditions, and self-reported practices are related in ENI management.

Therefore, this study examined ENI management among ICU healthcare professionals, most of whom were nurses, using a theory-informed approach. Accordingly, the findings primarily reflect the perceived knowledge, attitudes, and self-reported practices of ICU nurses. Specifically, we sought to: (1) assess knowledge, attitudes, and self-reported practices regarding ENI management and identify areas of relative weakness; (2) examine the relationships among the KAP domains and the associations of training and related factors with each domain; and (3) construct an observed-variable path model to examine the direct and indirect associations among knowledge, attitudes, and self-reported practices, with particular attention to the potential intermediary role of attitudes. By examining these relationships, this study extends previous descriptive evidence and may inform the design and prospective evaluation of targeted interventions. It was not intended to evaluate actual ENI management performance or clinical outcomes.

## 2 Methods

### 2.1 Study design

This study was a multicenter cross-sectional questionnaire survey designed to assess the levels of KAP regarding ENI management among ICU healthcare professionals and to explore the interrelationships among these three domains.

### 2.2 Participants

This study was conducted from December 2025 to February 2026. ICU healthcare professionals from 14 hospitals in Shandong and Henan provinces, China, were invited to participate. The inclusion criteria were as follows: (1) aged ≥18 years; (2) holding a valid professional license; (3) currently working in an ICU; (4) able to understand and independently complete the questionnaire; and (5) providing informed consent and voluntarily agreeing to participate. Participants were excluded if they were on leave, rotation, or training, were unable to participate for health-related reasons, or declined to take part in the study.

After data cleaning, 23 invalid questionnaires were excluded, including seven with incomplete responses, three duplicate submissions, and 13 questionnaires with a completion time of < 200 s. The final analytic sample comprised 478 complete questionnaires, with an effective completion rate of 95.4%. The participant screening process is shown in Supplementary Figure S1. Sample size was determined by the number of eligible ICU healthcare professionals recruited from the 14 participating hospitals during the predefined study period. No outcome-based *a priori* power calculation was performed. A sensitivity analysis using G^*^Power (Heinrich Heine University Düsseldorf, Düsseldorf, Germany) indicated that, with α = 0.05, power = 0.80, *N* = 478, and up to 10 predictor terms, the regression analyses could detect an overall effect size of approximately *f*^2^ = 0.035.

### 2.3 Ethical considerations

This study was approved by the Ethics Committee of the Affiliated Hospital of Shandong Second Medical University (approval No. wyfy-2025-ky-039) and was conducted in accordance with the Declaration of Helsinki and applicable institutional requirements. Before completing the questionnaire, all participants reviewed the study information online and provided electronic informed consent. Participation was voluntary and anonymous, and participants could withdraw at any time without adverse consequences.

### 2.4 Instruments

#### 2.4.1 General information

Basic information collected included gender, age, occupation, professional title, educational level, hospital type, years of ICU work experience, whether the participant had received EN-related training, and whether the participant was a member of an EN team. EN-related training was recorded as a self-reported dichotomous variable (yes/no). No information was collected on the training content, duration, frequency, delivery format, provider, or recency. Only hospital type was recorded; the specific hospital name or an anonymized hospital identifier was not collected.

#### 2.4.2 Knowledge-attitude-practice scale for ENI among ICU healthcare professionals

The Knowledge, Attitude and Practice Scale for ENI among ICU Healthcare Professionals, developed and validated by Mi et al. (12), comprises 41 items across three domains: knowledge (14 items), attitude (10 items), and practice (17 items). All items are rated on a five-point Likert scale, ranging from 1 (“not at all familiar”) to 5 (“completely familiar”) for knowledge, from 1 (“strongly disagree”) to 5 (“strongly agree”) for attitude, and from 1 (“never”) to 5 (“always”) for practice. Unweighted item scores are summed to yield domain scores of 14–70 for knowledge, 10–50 for attitude, and 17–85 for practice, with a total score ranging from 41 to 205. All items are scored in the same direction, and no reverse coding is required. Higher scores indicate greater ENI-related knowledge, more positive attitudes, and more frequent appropriate self-reported practices. These scores reflect participants' perceptions and reported practice tendencies rather than objectively verified knowledge or directly observed bedside performance. Because no validated cutoff values were applied, domain scores were analyzed as continuous variables and interpreted relative to their possible ranges rather than categorized as low, moderate, or high. The scale covers the definition and consequences of ENI, influencing factors, indications for feeding suspension, nutritional management strategies, and feeding-tolerance monitoring. The original instrument demonstrated good reliability and validity, with a Cronbach's α of 0.953, test-retest reliability of 0.795–0.977, item-level content validity indices of 0.800–1.000, and a scale-level content validity index of 0.975. In this study, Cronbach's α coefficients were 0.968 for knowledge, 0.988 for attitude, 0.973 for practice, and 0.982 for the overall scale. The complete questionnaire, response options, and scoring instructions are provided in Supplementary Appendix S1.

### 2.5 Data collection

Data were collected through an electronic questionnaire distributed via Wenjuanxing, a widely used online survey platform in China that enables efficient and standardized data collection among healthcare professionals. Before data collection, trained research coordinators were responsible for distributing the questionnaire and providing participants with standardized instructions to ensure accurate understanding of the survey content. All coordinators completed standardized training covering the study objectives, eligibility criteria, and questionnaire distribution procedures.

### 2.6 Statistical analysis

Statistical analyses were performed using IBM SPSS Statistics version 26.0 (IBM Corp., Armonk, NY, USA) and AMOS version 26.0 (IBM Corp., Armonk, NY, USA). Categorical variables were summarized as frequencies and percentages, and continuous variables as means and standard deviations. Because the knowledge, attitude, and practice scores did not fully conform to normal distributions, the Mann–Whitney U-test was used for comparisons between two groups, the Kruskal–Wallis H-test for comparisons among three or more groups, and Spearman's rank correlation analysis for associations between variables. Multiple linear regression models were constructed separately for knowledge, attitude, and practice scores. Variables showing statistically significant associations with the corresponding domain in the univariate analyses (*p* < 0.05) were entered into the respective multivariable models. Accordingly, the knowledge model included EN-related training, EN team membership, and hospital type; the attitude model included gender, EN-related training, EN team membership, and hospital type; and the practice model included age group, EN-related training, EN team membership, and hospital type. All predictors were entered simultaneously using the Enter method. Multicategory categorical variables were represented by k−1 dummy variables and entered as complete sets into the corresponding models. The reference categories were male, age ≤ 25 years, no EN-related training, no EN team membership, and tertiary Grade A hospital. The original composite scores were retained without transformation. Regression assumptions were assessed using residual vs. predicted plots, residual histograms, normal P–P plots, Durbin–Watson statistics, tolerance and variance inflation factors (VIFs), standardized residuals, studentized deleted residuals, leverage values, and Cook's distance. Observations with absolute standardized residuals >3 were further examined in sensitivity analyses by refitting the corresponding models after temporary exclusion. Although hospital type was considered in the regression analyses, specific hospital identifiers were not collected; therefore, within-hospital clustering could not be formally evaluated using multilevel models or cluster-adjusted sensitivity analyses. The final analytic dataset comprised 478 complete cases, with no missing values in the variables analyzed; therefore, no imputation was performed. All tests were two-sided, and *p* < 0.05 was considered statistically significant.

Based on the knowledge–attitude–practice framework and the preceding regression analyses, the observed-variable path model was specified before estimation. Knowledge, attitude, and practice domain total scores were entered as observed composite variables; no latent variables, item-level indicators, or measurement model were specified. The model included specified direct paths from knowledge to attitudes and self-reported practices and from attitudes to self-reported practices. Demographic, training-related, and organizational variables were included as observed exogenous variables only when supported by the regression findings and a clear outcome-specific theoretical or clinical rationale; variables lacking both were excluded to maintain model parsimony. Categorical variables were dummy-coded where appropriate. Modification indices and statistical significance were not used to add, remove, or redirect paths, and all prespecified paths were retained. Their unstandardized and standardized estimates are reported in Supplementary Table S5. Direct and indirect associations were estimated simultaneously, and the indirect association between knowledge and self-reported practice through attitudes was assessed using 5,000 bootstrap resamples with 95% confidence intervals. Model fit was evaluated using the chi-square statistic (χ^2^), the comparative fit index (CFI), the standardized root mean square residual (SRMR), and the root mean square error of approximation (RMSEA). Given the cross-sectional design, path coefficients and indirect associations were interpreted as features of the observed covariance structure rather than evidence of temporal ordering or causality.

## 3 Results

### 3.1 Participant characteristics

A total of 478 ICU healthcare professionals from 14 hospitals in Shandong and Henan provinces, China, were included in this study. Of these, 71.1% were female and 53.7% were aged 26–35 years. Nurses comprised 93.5% of the sample, and 93.9% had a bachelor's degree or higher. Junior and intermediate professional titles were held by 57.5% and 38.1% of participants, respectively. Nearly half had worked in the ICU for 0–5 years, while 25.1% had 6–10 years of ICU experience. In addition, 79.9% had received EN-related training and 25.5% were members of an EN team.

### 3.2 Univariate analysis of knowledge, attitude, and practice scores among ICU healthcare professionals

The mean item scores were 4.11 for self-assessed knowledge, 4.46 for attitude, and 4.40 for self-reported practice. Thus, the mean item score for self-assessed knowledge was comparatively lower than those for attitude and self-reported practice. The lowest self-assessed knowledge scores were observed for the two items concerning the operational definitions of continuous and intermittent ENI. Item-level descriptive statistics are presented in Supplementary Table S1–S3. The univariate analysis showed that training-related factors demonstrated the most consistent and significant associations across all three domains (Table 1; Figure 1). Compared with participants who had not received training, those who had received training had higher knowledge, attitude, and practice scores (*U* = 9656.5, 13846.5, and 12886.5, respectively; all *p* < 0.01). Similarly, EN team members scored higher than non-members across the knowledge, attitude, and practice dimensions (*U* = 15122.5, *p* < 0.01; U = 18936.5, *p* < 0.05; and U = 15117.5, *p* < 0.01, respectively) (Figure 2).

**Table 1:** Baseline characteristics of ICU healthcare professionals and univariate analysis of knowledge, attitude, and practice scores.

Category	Number (%)	Knowledge score, mean ±SD/statistic	Attitude score, mean ±SD/statistic	Practice score, mean ±SD/statistic
**Gender**		U = 23086.5	U = 20593^*^	U = 21564
Male	138 (28.9%)	56.94 ± 12.198	43.49 ± 8.366	73.26 ± 12.777
Female	340 (71.1%)	57.71 ± 11.314	45.04 ± 7.295	75.34 ± 11.331
**Age**		H = 7.662	H = 8.444	H = 11.02^*^
≤25 years	89 (18.6%)	55.17 ± 11.48	43.36 ± 7.98	71.45 ± 13.14
26–30 years	166 (34.7%)	58.19 ± 11.32	45.13 ± 7.18	76.20 ± 11.04
31–35 years	91 (19.0%)	59.08 ± 11.00	45.01 ± 7.39	75.34 ± 11.10
36–40 years	93 (19.5%)	56.73 ± 11.53	43.84 ± 7.97	74.44 ± 12.10
≥41 years	39 (8.2%)	57.87 ± 13.72	45.97 ± 8.40	75.31 ± 11.60
**Occupation**		U = 6914.5	U = 6650	U = 6787.5
Physician	31 (6.5%)	57.03 ± 13.17	45.13 ± 6.95	74.19 ± 13.05
Nurse	447 (93.5%)	57.52 ± 11.47	44.56 ± 7.70	74.78 ± 11.71
**Professional title**		H = 1.837	H = 2.823	H = 0.717
Junior	275 (57.5%)	57.29 ± 11.57	44.30 ± 7.64	74.76 ± 11.91
Intermediate	182 (38.1%)	57.54 ± 11.44	44.88 ± 7.64	74.68 ± 11.37
Associate senior	18 (3.8%)	60.78 ± 12.73	46.44 ± 7.59	75.78 ± 14.02
Senior	3 (0.6%)	52.67 ± 15.14	43.00 ± 11.27	70.33 ± 17.47
**Educational level**		H = 2.765	H = 1.634	H = 5.285
Junior college	29 (6.1%)	58.17 ± 12.60	43.72 ± 8.96	76.93 ± 12.21
Bachelor's degree	424 (88.7%)	57.65 ± 11.40	44.67 ± 7.65	74.75 ± 11.77
Master's degree	25 (5.2%)	53.92 ± 12.97	44.36 ± 6.01	72.08 ± 11.68
**ICU work experience**		H = 2.053	H = 1.485	H = 4.134
0–5 years	233 (48.7%)	57.15 ± 11.48	44.64 ± 7.49	74.18 ± 12.18
6–10 years	120 (25.1%)	58.23 ± 11.37	44.73 ± 7.17	75.83 ± 11.17
11–15 years	88 (18.4%)	58.11 ± 11.15	44.83 ± 7.79	75.09 ± 11.51
16–20 years	28 (5.9%)	54.75 ± 13.77	42.71 ± 10.08	72.18 ± 12.69
≥21 years	9 (1.9%)	58.56 ± 14.16	45.33 ± 8.72	79.11 ± 8.55
**Participation in EN-related training**		U = 9656.5^**^	U = 13846.5^**^	U = 12886.5^**^
Yes	382 (79.9%)	59.54 ± 10.30	45.36 ± 7.07	76.15 ± 10.66
No	96 (20.1%)	49.30 ± 12.73	41.56 ± 9.03	69.13 ± 14.24
**EN team membership**		U = 15122.5^**^	U = 18936.5^*^	U = 15117.5^**^
Yes	122 (25.5%)	61.58 ± 11.05	45.85 ± 7.29	78.66 ± 9.81
No	356 (74.5%)	56.08 ± 11.42	44.16 ± 7.72	73.40 ± 12.12
**Hospital type**		H = 12.81^*^	H = 12.60^*^	H = 30.34^**^
Tertiary Grade A hospital	255 (53.3%)	57.04 ± 10.48	44.45 ± 7.12	73.15 ± 11.88
Tertiary Grade B hospital	101 (21.1%)	57.57 ± 12.29	45.86 ± 7.95	77.43 ± 10.14
Tertiary hospital	12 (2.5%)	56.25 ± 13.67	43.08 ± 7.90	76.67 ± 8.90
Secondary Grade A hospital	106 (22.2%)	59.25 ± 12.61	44.24 ± 8.28	76.72 ± 11.73
Other	4 (0.8%)	40.25 ± 12.55	36.00 ± 9.52	49.75 ± 13.60

**Note:** Values are presented as mean ± standard deviation unless otherwise indicated. ^*^p < 0.05 and ^**^p < 0.01 indicate statistical significance. U denotes the Mann–Whitney U test, and H denotes the Kruskal–Wallis H-test.

**Figure 1:**
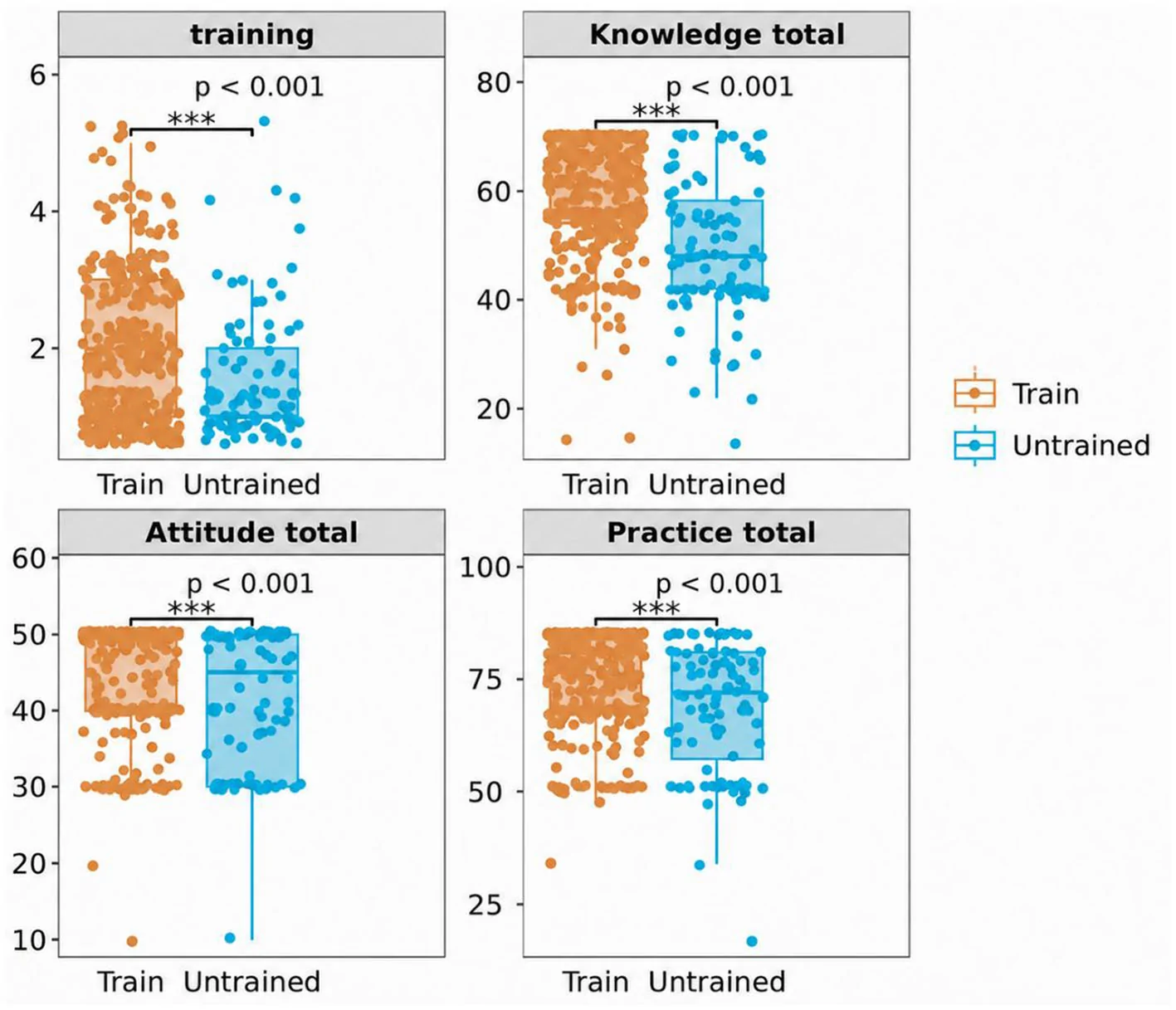
Univariate comparison of knowledge, attitude, and practice scores for ENI management according to training experience among ICU healthcare professionals. ^***^p < 0.001.

**Figure 2:**
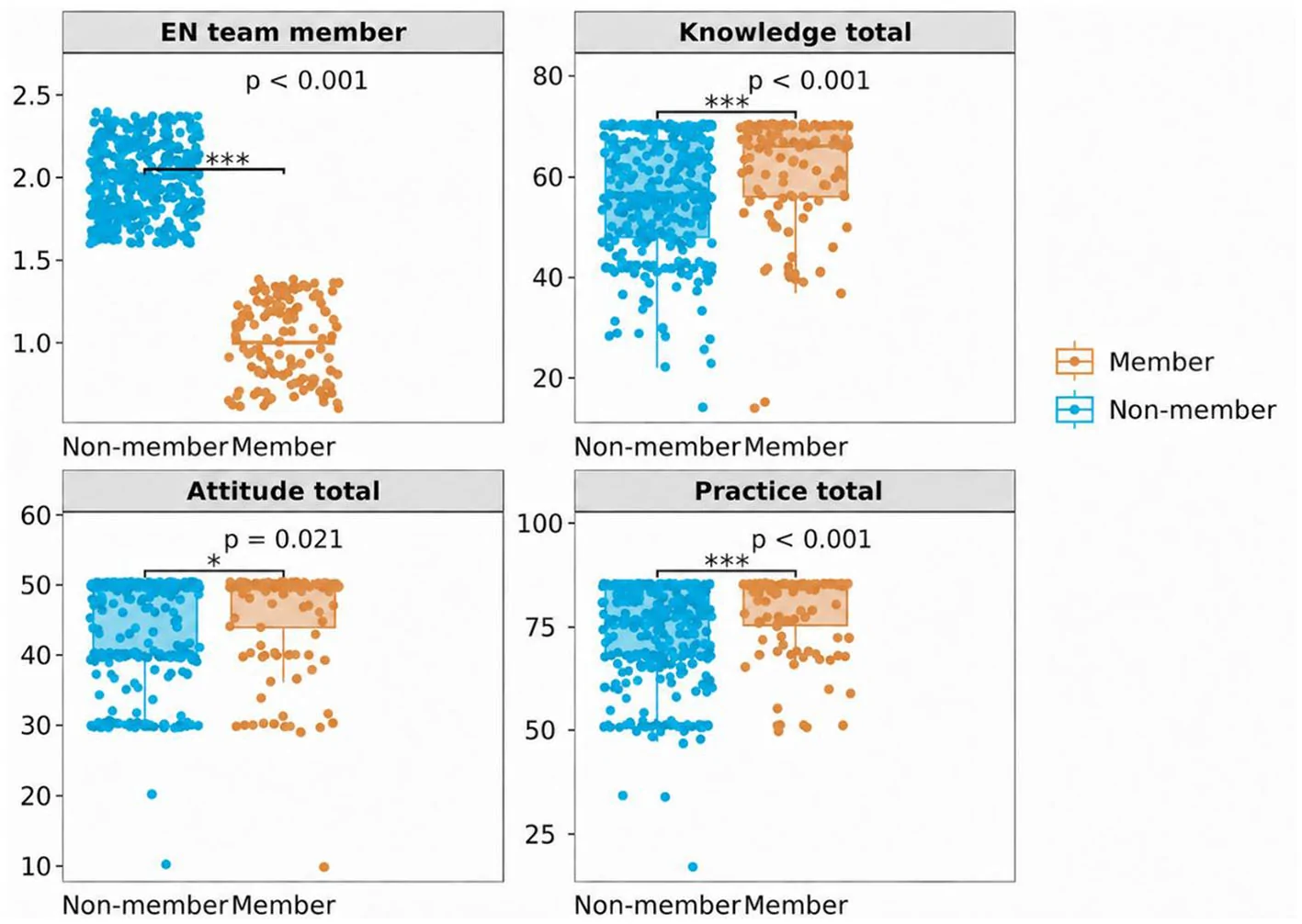
Univariate comparison of knowledge, attitude, and practice scores for ENI management according to EN team membership among ICU healthcare professionals. ^*^p < 0.05 and ^***^p < 0.001.

In contrast, demographic variables showed more limited and domain-specific associations. Age was significantly associated only with practice scores (*p* < 0.05), while gender differences were observed only in the attitude domain, with female participants scoring higher than male participants (*p* < 0.05). Hospital type was associated with all three domains (*p* < 0.05), although the magnitude of these differences was smaller than that observed for training-related variables. No statistically significant differences were found according to occupation, professional title, educational level, or ICU work experience (all *p* > 0.05) (Table 1).

### 3.3 Correlation analysis of knowledge, attitudes, and practices

Spearman correlation analysis showed significant positive correlations among knowledge, attitude, and practice scores (all *p* < 0.001). The strongest correlation was observed between knowledge and practice (ρ = 0.738), followed by attitude and practice (ρ = 0.722), and knowledge and attitude (ρ = 0.692) (Table 2).

**Table 2:** Spearman correlation analysis of knowledge, attitude, and practice scores

Variable Pair	Spearman Correlation (ρ)	*p*-value
Knowledge score vs. attitude score	0.692	<0.001
Attitude score vs. practice score	0.722	<0.001
Knowledge score vs. practice score	0.738	<0.001

### 3.4 Multiple linear regression analysis of factors associated with knowledge, attitude, and practice scores

Multiple linear regression analyses identified several factors associated with knowledge, attitude, and practice scores among ICU healthcare professionals. All three models were statistically significant (knowledge: *F* = 14.088; attitude: *F* = 4.893; practice: *F* = 7.898; all *p* < 0.001), although they explained a modest proportion of the variance in the outcomes (adjusted *R*^2^ = 0.054–0.141). Participation in EN-related training was positively associated with knowledge (β = 0.315, *p* < 0.001), attitude (β = 0.185, *p* < 0.001), and practice scores (β = 0.193, *p* < 0.001). EN team membership was positively associated with knowledge (β = 0.112, *p* = 0.013) and practice scores (β = 0.136, *p* = 0.003). Female participants had higher attitude scores than male participants (β = 0.098, *p* = 0.030), while participants aged 26–30 years had higher practice scores than those aged ≤ 25 years (β = 0.147, *p* = 0.015). Compared with participants from tertiary Grade A hospitals, those from tertiary Grade B hospitals had higher practice scores (β = 0.147, *p* = 0.002), whereas participants from hospitals classified as “other” had lower knowledge (β = −0.087, *p* = 0.044) and practice scores (β = −0.155, *p* < 0.001). VIF values ranged from approximately 1.02 to 1.98, indicating no problematic multicollinearity. Only statistically significant coefficients are shown in Table 3; nonsignificant coefficients are provided in Supplementary Table S6.

**Table 3:** Multivariable linear regression analysis of factors associated with knowledge, attitude, and practice scores.

Model	Associated factor	*B*	SE	β	*t*	*p*	VIF	Adjusted *R^2^*	*F*	*p*
**Knowledge**	EN-related training (yes vs. no)	9.083	1.284	0.315	7.074	<0.001	1.10	0.141	14.088	<0.001
	EN team membership (yes vs. no)	2.980	1.200	0.112	2.483	0.013	1.14			
	Hospital type (other vs. tertiary Grade A hospital)	−10.978	5.448	−0.087	−2.015	0.044	1.02			
**Attitude**	Gender (female vs. male)	1.653	0.758	0.098	2.181	0.030	1.02	0.054	4.893	<0.001
	EN-related training (yes vs. no)	3.524	0.892	0.185	3.951	<0.001	1.10			
**Practice**	EN-related training (yes vs. no)	5.685	1.328	0.193	4.281	<0.001	1.11	0.126	7.898	<0.001
	EN team membership (yes vs. no)	3.682	1.236	0.136	2.979	0.003	1.15			
	Age group (26–30 years vs. ≤ 25 years)	3.645	1.490	0.147	2.446	0.015	1.98			
	Hospital type (tertiary Grade B vs. tertiary Grade A hospital)	4.239	1.328	0.147	3.192	0.002	1.16			
	Hospital type (other vs. tertiary Grade A hospital)	−20.080	5.661	−0.155	−3.547	<0.001	1.05			

**Note:** *B* = unstandardized regression coefficient; SE = standard error of the unstandardized coefficient; β = standardized regression coefficient; VIF = variance inflation factor. Only statistically significant coefficients from the multivariable models are presented; nonsignificant coefficients are reported in Supplementary Table S6. Reference categories were male, age ≤ 25years, no EN-related training, no EN team membership, and tertiary Grade A hospital.

Sensitivity analyses yielded generally similar model fit and coefficient directions. The principal associations in the knowledge and practice models remained broadly stable, whereas some coefficients in the attitude model, particularly those involving gender and hospital-category contrasts, showed greater sensitivity to the exclusion of observations with large standardized residuals. These estimates were therefore interpreted cautiously. Detailed diagnostic and sensitivity-analysis results are provided in Supplementary Table S4.

### 3.5 Observed-variable path analysis of the relationships among knowledge, attitudes, and practices

The observed-variable path model showed good overall fit to the data (χ^2^ (15) = 22.710, χ^2^*/*df = 1.514, CFI = 0.994, SRMR = 0.024, and RMSEA = 0.033), supporting the compatibility of the specified path structure with the observed covariance matrix. Knowledge was positively associated with attitudes, and both knowledge and attitudes were associated with practices. Among the external factors, training was primarily associated with knowledge, EN team membership with both knowledge and practices, and hospital-related differences were more evident for practices; gender showed a comparatively limited association with attitudes. Detailed standardized coefficients for the statistically significant paths are shown in Figure 3, while complete estimates for all specified paths, including nonsignificant paths, are provided in Supplementary Table S5. A separate bootstrap analysis with 5,000 resamples indicated a significant indirect association between knowledge and practices through attitudes (indirect effect = 0.339, 95% CI: 0.246–0.438, *p* < 0.001), suggesting a potential intermediary role of attitudes. Given the cross-sectional design, these estimates describe observed associations and do not establish temporal ordering or causal direction.

**Figure 3:**
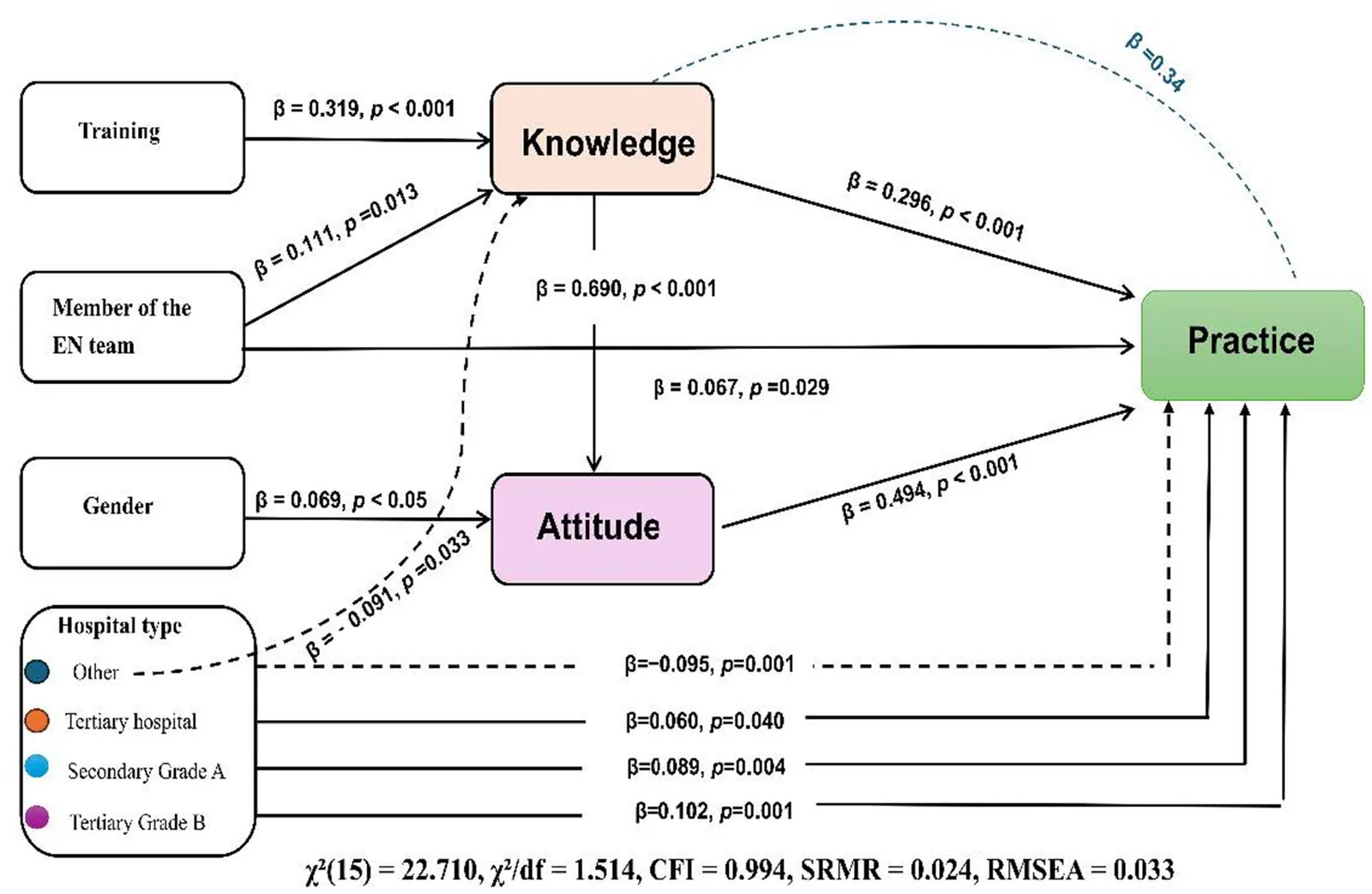
Observed-variable path analysis of knowledge, attitude, and practice domain scores **Note:** Knowledge, attitude, and practice were modeled as observed composite scores. The figure presents standardized coefficients for statistically significant paths; complete estimates for all specified paths are provided in Supplementary Table S5. Solid black, dashed black, and dashed blue lines indicate positive direct, negative direct, and indirect paths, respectively.

## 4 Discussion

This multicenter cross-sectional study found that ICU healthcare professionals, predominantly nurses, reported comparatively high mean item scores for attitudes and self-reported practices toward ENI management, while knowledge was comparatively weaker, particularly regarding the operational definitions of continuous and intermittent ENI. The three KAP domains were positively associated, and attitudes showed a possible intermediary role in the relationship between knowledge and practice. EN-related training was consistently associated with higher scores across all domains, whereas EN team membership was associated with higher knowledge and practice scores. Associations with gender, age, and hospital type were less consistent. The modest explanatory power of the regression models suggests that ENI-related KAP may also be influenced by unmeasured organizational and workflow factors.

The comparatively lower knowledge score should be interpreted as a relative weakness in self-assessed knowledge rather than evidence of objectively demonstrated inadequate knowledge or clinical competence. Participants reported favorable attitudes and frequent appropriate self-reported practices, yet their self-assessed familiarity with the operational criteria for identifying ENI was less consistent. This pattern is broadly consistent with previous studies of EN management among ICU nurses. Song et al. (15) reported less favorable knowledge than attitude and practice scores, while Ni et al. (16) and Zare-Kaseb et al. (17) identified variation in nurses' EN-related knowledge and practices and highlighted inadequate training and limited awareness of standardized protocols as important barriers. The present study extends this evidence by focusing specifically on ENI, which requires timely and context-sensitive decisions about whether feeding should be suspended, when it should be resumed, and how missed nutritional delivery should be addressed after procedures, airway management, gastrointestinal intolerance, feeding-tube problems, or hemodynamic changes (6, 14, 22–25). Because these decisions are embedded in complex ICU workflows, specific knowledge gaps may coexist with apparently appropriate practices when bedside care is guided by medical orders, established routines, local protocols, or team experience. This interpretation is consistent with evidence that ENI is shaped not only by patients' clinical conditions but also by procedure scheduling, communication, workflow barriers, and delays in restarting feeding (6, 7, 9–11, 23–25). The positive associations among KAP are also compatible with the theoretical framework of this study. Knowledge Translation Theory emphasizes that evidence must be adapted to the clinical context, linked to identified barriers, and translated into feasible implementation strategies before it can influence practice (18, 20), whereas the Theory of Planned Behavior suggests that attitudes may connect knowledge with behavioral intentions and subsequent practice (19, 21). Accordingly, a clearer understanding of the consequences and management of ENI may strengthen healthcare professionals' recognition of its clinical importance and support more consistent practices. Nevertheless, the indirect association through attitudes should be viewed as a theory-informed interpretation of the observed relationships rather than confirmation of a temporal behavioral mechanism. The consistent association between EN-related training and all three KAP domains suggests that previous exposure to EN education may be relevant to participants' self-assessed knowledge, attitudes, and self-reported practices. However, training was recorded only as a yes/no variable, without information on its content, duration, frequency, format, or recency. The study therefore cannot identify which training characteristics may account for the observed associations or determine whether a particular educational approach would be effective. These findings should instead be viewed as supporting the prospective development and evaluation of clearly defined ENI-related educational interventions. Similarly, the association of EN team membership with higher knowledge and practice scores may reflect greater involvement in nutritional assessment, care planning, bedside consultation, and quality-improvement activities. Previous studies have likewise emphasized the importance of multidisciplinary collaboration and standardized protocols in addressing process-related barriers to EN delivery (9–11, 24). By contrast, the associations with gender and age were small and not fully stable across analyses and should therefore be considered exploratory. Hospital-related differences were more evident for self-reported practices than for the other KAP domains. These differences may reflect variation in local protocols, staffing, nutritional support resources, or workflow organization across hospital settings, although these contextual factors were not measured directly. The hospital-specific estimates should therefore be interpreted cautiously.

From a clinical and organizational perspective, the relatively lower self-assessed knowledge of ENI operational definitions suggests a need for greater clarity and consistency in how ENI is recognized and managed in routine ICU practice. The associations of EN-related training with all three KAP domains, and of EN team membership with knowledge and practice, further underscore the relevance of structured education and multidisciplinary support. In practice, greater emphasis may be placed on clarifying when EN interruption is warranted, supporting timely resumption of feeding, and standardizing responses to common interruption scenarios such as gastrointestinal intolerance, feeding-tube-related problems, and hemodynamic instability. These implications should be interpreted cautiously, as the study assessed self-reported KAP rather than actual ENI management or patient outcomes. Several limitations should also be considered when interpreting these findings. First, the cross-sectional design precludes causal inference and does not establish the temporal sequence among KAP. Second, all three KAP domains were assessed using a self-administered questionnaire: knowledge scores reflected self-assessed familiarity rather than objectively tested competence, whereas practice scores represented reported tendencies rather than directly observed bedside performance. Third, EN-related training was recorded only as a self-reported yes/no variable, without details on its content, duration, frequency, format, or recency, limiting our ability to identify relevant training characteristics or recommend a specific educational approach. Fourth, objective indicators of ENI management, including interruption frequency and duration, time to feeding resumption, caloric or protein adequacy, and patient outcomes, were not collected; therefore, the observed associations cannot establish whether higher KAP scores, previous training, or EN team membership translate into better clinical performance or outcomes. Fifth, the path analysis used observed composite scores rather than latent variables, so measurement error was not explicitly modeled. The estimated paths represent statistical associations rather than a confirmed behavioral mechanism, and the favorable fit indices do not establish that the proposed structure is the only plausible model. Sixth, the predominance of nurses and recruitment from 14 hospitals in two provinces limit profession-specific comparisons and may restrict generalizability to the broader multidisciplinary ICU workforce and other settings. In addition, some hospital-type categories contained very small numbers of participants, particularly the “other” category (*n* = 4) and the tertiary hospital category (*n* = 12); therefore, hospital-specific estimates may be unstable and should be regarded as exploratory. Finally, the modest explanatory power of the regression models suggests that relevant organizational and workflow-related factors were not fully captured, while the absence of anonymized hospital identifiers prevented assessment of within-hospital clustering. Future longitudinal and interventional studies should therefore recruit more diverse professional and institutional samples, retain hospital-level identifiers, characterize educational exposure in greater detail, incorporate objective ENI process, nutritional adequacy, and patient outcome measures, and use multilevel and latent-variable approaches to clarify the robustness, temporal sequence, and clinical relevance of these relationships.

## 5 Conclusion

Among this predominantly nursing cohort, ENI-related knowledge, attitudes, and self-reported practices were positively associated, with attitudes showing a possible intermediary role in the relationship between knowledge and practice. EN-related training was associated with more favorable scores across all three KAP domains, while EN team membership was associated with higher knowledge and practice scores, highlighting potential targets for future educational and organizational strategies. However, given the cross-sectional, questionnaire-based design, these associations should not be interpreted as evidence of improved ENI management or patient outcomes. Prospective longitudinal and interventional studies incorporating objective ENI process measures, nutritional adequacy, and patient outcomes are needed to clarify the temporal and clinical significance of these relationships.

## Data Availability

The raw data supporting the conclusions of this article will be made available by the authors, without undue reservation.
