# Supplementary material for: Knowledge, attitudes, and practices regarding enteral nutrition interruption management in intensive care units: a multicenter cross-sectional study predominantly among nurses
Source: Front Nutr. 2026 Oct 2;13:1888995. doi: 10.3389/fnut.2026.1888995 (PMC13671998; doi:10.3389/fnut.2026.1888995)
Supplement: Supplementary file 1 [file Supplementary_file_1.docx]

**Questionnaire on ICU Healthcare Professionals’ Knowledge, Attitudes, and Practices Regarding ENI Management**

**(I) General Information**

1. Your gender: □ Female; □ Male
2. Your age: □ ≤25 years; □ 26–30 years; □ 31–35 years; □ 36–40 years; □ ≥41 years
3. Job Type：□ Doctor; □Nurse
4. Professional Title: □Junior; □Intermediate; □ Associate Senior; □ Senior
5. Educational Level: □Junior College; □Bachelor’s Degree □Master’s Degree; □Doctoral Degree
6. Years of ICU Work Experience:
7. Have you received training related to enteral nutrition: □Yes; □No
8. Are you a member of the enteral nutrition team: □Yes; □No
9. Hospital type:

**(II) Knowledge Section**

1. Under continuous enteral nutrition infusion, an interruption lasting ≥1 hour is defined as an enteral nutrition interruption.

A. Not at all familiar; B. Uncertain; C. Slightly familiar; D. Moderately familiar ; E. Completely familiar

2. Under intermittent enteral nutrition infusion (three times per day, each lasting 30 minutes), failure to deliver the prescribed nutritional volume within 30 minutes is defined as an enteral nutrition interruption.

A. Not at all familiar; B. Uncertain; C. Slightly familiar; D. Moderately familiar ; E. Completely familiar

3. Once enteral nutrition interruption occurs, it may affect patients’ energy target achievement and increase the risk of malnutrition.

A. Not at all familiar; B. Uncertain; C. Slightly familiar; D. Moderately familiar ; E. Completely familiar

4. Enteral nutrition interruption is positively associated with disease severity, hospitalization costs, and target caloric requirements.

A. Not at all familiar; B. Uncertain; C. Slightly familiar; D. Moderately familiar ; E. Completely familiar

5. Hemodynamic instability, increased intra-abdominal pressure, and gastrointestinal complications (e.g., intestinal obstruction, anastomotic leakage, chylothorax) are influencing factors of enteral nutrition interruption.

A. Not at all familiar; B. Uncertain; C. Slightly familiar; D. Moderately familiar ; E. Completely familiar

6. Medical and nursing procedures (e.g., general anesthesia surgery, radiological examinations, bronchoscopy, artificial airway establishment/replacement, repositioning, suctioning) are causes of enteral nutrition interruption.

A. Not at all familiar; B. Uncertain; C. Slightly familiar; D. Moderately familiar ; E. Completely familiar

7. Difficult tube placement, feeding tube obstruction, displacement, or dislodgement in critically ill patients can lead to feeding interruption.

A. Not at all familiar; B. Uncertain; C. Slightly familiar; D. Moderately familiar ; E. Completely familiar

8. For critically ill patients with stable hemodynamics and normal gastrointestinal function, enteral nutrition should be initiated early within 24–48 hours after ICU admission.

A. Not at all familiar; B. Uncertain; C. Slightly familiar; D. Moderately familiar ; E. Completely familiar

9. For bolus or intermittent gravity feeding, gastric residual volume should be assessed before each feeding; for continuous pump feeding, it should be assessed at least every 4 hours.

A. Not at all familiar; B. Uncertain; C. Slightly familiar; D. Moderately familiar ; E. Completely familiar

10. Sedative and analgesic drugs may impair gastrointestinal motility and delay gastric emptying; therefore, pain and sedation levels should be dynamically assessed, and drug use minimized whenever possible.

A. Not at all familiar; B. Uncertain; C. Slightly familiar; D. Moderately familiar ; E. Completely familiar

11. Prokinetic agents can improve gastrointestinal intolerance symptoms in critically ill patients.

A. Not at all familiar; B. Uncertain; C. Slightly familiar; D. Moderately familiar ; E. Completely familiar

12. For critically ill patients with elevated intra-abdominal pressure (IAP > 12 mmHg), routine monitoring should be established, and enteral nutrition delivery should be adjusted accordingly.

A. Not at all familiar; B. Uncertain; C. Slightly familiar; D. Moderately familiar ; E. Completely familiar

13. During enteral nutrition, the head of the bed should be elevated to 30°–45° unless contraindicated.

A. Not at all familiar; B. Uncertain; C. Slightly familiar; D. Moderately familiar ; E. Completely familiar

14. Enteral nutrition should be resumed promptly after medical or nursing procedures are completed.

A. Not at all familiar; B. Uncertain; C. Slightly familiar; D. Moderately familiar ; E. Completely familiar

**(III) Attitude Section**

1. I believe that it is very important for ICU nurses to master knowledge related to enteral nutrition interruption.

A. Strongly disagree; B. Disagree; C. Neutral; D. Agree; E. Strongly agree

2. I believe that hospitals (or departments) should provide formal training on enteral nutrition tolerance.

A. Strongly disagree; B. Disagree; C. Neutral; D. Agree; E. Strongly agree

3. I believe that having more knowledge about enteral nutrition interruption is highly beneficial to my clinical practice.

A. Strongly disagree; B. Disagree; C. Neutral; D. Agree; E. Strongly agree

4. I believe that nutritional assessment is very important for all ICU patients.

A. Strongly disagree; B. Disagree; C. Neutral; D. Agree; E. Strongly agree

5. I believe that nutritional assessment of ICU patients requires multidisciplinary collaboration between physicians and nurses.

A. Strongly disagree; B. Disagree; C. Neutral; D. Agree; E. Strongly agree

6. I believe that selecting appropriate enteral nutrition formulas is very important for preventing enteral nutrition interruption.

A. Strongly disagree; B. Disagree; C. Neutral; D. Agree; E. Strongly agree

7. I believe that establishing and selecting appropriate enteral nutrition feeding routes is important.

A. Strongly disagree; B. Disagree; C. Neutral; D. Agree; E. Strongly agree

8. I believe that patient positioning during enteral nutrition is very important.

A. Strongly disagree; B. Disagree; C. Neutral; D. Agree; E. Strongly agree

9. I believe that standardized enteral nutrition management protocols are essential for preventing and managing feeding interruption.

A. Strongly disagree; B. Disagree; C. Neutral; D. Agree; E. Strongly agree

10. I believe that prevention of enteral nutrition interruption is more important than treatment.

A. Strongly disagree; B. Disagree; C. Neutral; D. Agree; E. Strongly agree

**(IV) Practice Section**

1. I actively seek to learn knowledge related to enteral nutrition interruption.

A. Never; B. Rarely; C. Sometimes; D. Often; E. Always

2. I actively communicate with patients or their families about the importance of enteral nutrition and the risks of interruption.

A. Never; B. Rarely; C. Sometimes; D. Often; E. Always

3. Upon ICU admission, I promptly assess patients’ nutritional status and communicate with the responsible physician.

A. Never; B. Rarely; C. Sometimes; D. Often; E. Always

4. Before initiating enteral nutrition, I communicate with physicians to establish an appropriate feeding route.

A. Never; B. Rarely; C. Sometimes; D. Often; E. Always

5. I strictly adhere to hand hygiene during enteral nutrition administration.

A. Never; B. Rarely; C. Sometimes; D. Often; E. Always

6. In the absence of contraindications, I elevate the head of the bed to 30°–45° for patients receiving enteral nutrition.

A. Never; B. Rarely; C. Sometimes; D. Often; E. Always

7. During enteral nutrition, I monitor feeding intolerance symptoms (e.g., nausea, vomiting, reflux/aspiration, abdominal distension) and report them promptly.

A. Never; B. Rarely; C. Sometimes; D. Often; E. Always

8. I stop enteral nutrition when patients undergo emergency artificial airway establishment or replacement (e.g., intubation, tracheostomy).

A. Never; B. Rarely; C. Sometimes; D. Often; E. Always

9. I stop enteral nutrition during bedside X-ray or bronchoscopy procedures.

A. Never; B. Rarely; C. Sometimes; D. Often; E. Always

10. I stop enteral nutrition when patients deteriorate and require emergency surgery or are expected to undergo general anesthesia within 4–8 hours.

A. Never; B. Rarely; C. Sometimes; D. Often; E. Always

11. For patients with feeding intolerance, I assess causes and collaborate with physicians to determine whether enteral nutrition should be discontinued.

A. Never; B. Rarely; C. Sometimes; D. Often; E. Always

12. I stop enteral nutrition in patients with uncorrected shock, unstable hemodynamics, or inadequate tissue perfusion (e.g., MAP < 50 mmHg despite escalating vasopressors).

A. Never; B. Rarely; C. Sometimes; D. Often; E. Always

13. I stop enteral nutrition in patients with life-threatening hypoxemia, hypercapnia, or acidosis that is difficult to control.

A. Never; B. Rarely; C. Sometimes; D. Often; E. Always

14. I stop enteral nutrition in critically ill patients with active upper gastrointestinal bleeding or intestinal ischemia.

A. Never; B. Rarely; C. Sometimes; D. Often; E. Always

15. I stop enteral nutrition in patients with elevated intra-abdominal pressure (IAP > 20 mmHg).

A. Never; B. Rarely; C. Sometimes; D. Often; E. Always

16. During enteral nutrition, if gastric residual volume exceeds 250 mL on two consecutive assessments, I notify the physician to consider prokinetic agents.

A. Never; B. Rarely; C. Sometimes; D. Often; E. Always

17. For patients intolerant to gastric feeding despite prokinetics or at high risk of aspiration, I communicate with physicians to establish post-pyloric feeding access.
A. Never; B. Rarely; C. Sometimes; D. Often; E. Always

**Scoring instructions**

Scoring instructions: The instrument comprises 41 items across three domains: knowledge (14 items), attitude (10 items), and practice (17 items). Each item is scored on a five-point Likert scale. Knowledge items are scored from 1 (“not at all familiar”) to 5 (“completely familiar”); attitude items are scored from 1 (“strongly disagree”) to 5 (“strongly agree”); and practice items are scored from 1 (“never”) to 5 (“always”). Item scores are summed without weighting to obtain the corresponding domain scores. The possible score ranges are 14–70 for knowledge, 10–50 for attitude, and 17–85 for practice, with a total score ranging from 41 to 205. All items are scored in the same direction, and no reverse coding is required. Higher scores indicate greater knowledge of ENI management, more positive attitudes, and more frequent appropriate self-reported practices. No validated cutoff values were applied; therefore, the domain scores were treated as continuous variables and interpreted relative to their possible ranges rather than categorized as low, moderate, or high.
