# Supplementary material for: Knowledge, attitudes, and practices regarding enteral nutrition interruption management in intensive care units: a multicenter cross-sectional study predominantly among nurses
Source: Front Nutr. 2026 Oct 2;13:1888995. doi: 10.3389/fnut.2026.1888995 (PMC13671998; doi:10.3389/fnut.2026.1888995)
Supplement: Supplementary file 2 [file Supplementary_file_2.docx]

1. **Participant Characteristics**

Total questionnaires distributed: 501

a. 7 were incomplete,

b. 3 were duplicates, and

c. 13 were completed in under 200 seconds.

23 excluded responses

Final valid responses included in the analysis: 478

**Supplementary Figure S1** **Participant selection flowchart**
