# Supplementary material for: Knowledge, attitudes, and practices regarding enteral nutrition interruption management in intensive care units: a multicenter cross-sectional study predominantly among nurses
Source: Front Nutr. 2026 Oct 2;13:1888995. doi: 10.3389/fnut.2026.1888995 (PMC13671998; doi:10.3389/fnut.2026.1888995)
Supplement: Supplementary file 3 [file Table_1.docx]

**Supplementary Table S1. Item-level descriptive statistics for ENI-related knowledge scores**

| **Knowledge—Item** | | **Mean (SD)** |
| --- | --- | --- |
| **K1** | Under the condition of continuous enteral nutrition infusion, if the interruption of enteral nutrition lasts for 1 hour or more, it can be defined as an interruption of enteral nutrition. | 3.69 (1.16) |
| **K2** | Under the condition of intermittent enteral nutrition infusion, the infusion is carried out 3 times a day, each time lasting for 30 minutes. If the patient does not obtain the expected amount of nutrition within 30 minutes, it can be defined as an interruption of enteral nutrition. | 3.70 (1.14) |
| **K3** | Once enteral nutrition is interrupted, it may prevent patients from reaching their energy targets, thereby increasing the risk of nutritional deficiency for the patients. | 4.13 (0.97) |
| **K4** | The interruption of enteral nutrition is positively correlated with the severity of the patient's condition, hospitalization costs, and target calorie intake. | 4.04 (1.07) |
| **K5** | Hemodynamic instability, excessive intra-abdominal pressure, and digestive system-related complications, such as intestinal obstruction, anastomotic leakage, and chylothorax, are the influencing factors for the interruption of enteral nutrition. | 4.14 (0.96) |
| **K6** | Medical and nursing-related procedures such as general anesthesia surgeries, radiological examinations, endotracheal bronchoscopy, establishment/replacement of artificial airways, position changes, and suctioning are all reasons for the interruption of enteral nutrition. | 4.14 (0.96) |
| **K7** | Difficulties in catheterization for critically ill patients, blockages, displacements and detachments of nutritional infusion tubes are the causes of feeding interruptions. | 4.15 (0.93) |
| **K8** | For critically ill patients, if their gastrointestinal function is good and their hemodynamic stability is maintained within 24 to 48 hours after admission, early initiation of enteral nutrition should be carried out. | 4.25 (0.92) |
| **K9** | For critically ill patients receiving enteral nutrition through multiple bolus infusions or intermittent gravity drip feeding, the gastric residual volume should be monitored before each feeding; during continuous pump infusion, the gastric residual volume should be measured at least every 4 hours. | 4.23 (0.93) |
| **K10** | sedative and analgesic drugs can affect the gastrointestinal function of patients, causing delayed gastric emptying. It is necessary to dynamically assess the patient's pain level and the depth of sedation, and, when feasible within the treatment plan and the patient's wishes, minimize the use of sedative and analgesic drugs as much as possible. | 4.14 (0.96) |
| **K11** | Gastrointestinal motility-promoting drugs can alleviate the gastrointestinal intolerance symptoms in critically ill patients. | 4.16 (0.94) |
| **K12** | For critically ill patients with elevated intra-abdominal pressure (IAP > 12 mmHg), it is necessary to establish a routine for intra-abdominal pressure monitoring, and adjust the infusion rate and total volume of enteral nutrition according to the intra-abdominal pressure level. | 4.11 (0.97) |
| **K13** | During the period of enteral nutrition for critically ill patients, apart from any special contraindications, the head of the bed should be elevated to 30° to 45°. | 4.32 (0.89) |
| **K14** | After all the medical and nursing-related examination and treatment procedures are completed, it is necessary to promptly resume enteral nutrition feeding. | 4.29 (0.91) |

**Supplementary Table S2. Item-level descriptive statistics for ENI-related attitude scores**

| **Attitude—Item** | | **Mean (SD)** |
| --- | --- | --- |
| **A1** | I believe that ICU nurses need to have a thorough understanding of the relevant knowledge regarding enteral nutrition interruptions. | 4.46 (0.82) |
| **A2** | I believe that hospitals (or departments) should conduct formal training on the knowledge of enteral nutrition tolerance. | 4.45 (0.81) |
| **A3** | I believe that having a good understanding of more aspects related to enteral nutrition interruptions is of great help to my clinical work. | 4.43 (0.82) |
| **A4** | I believe it is extremely important to conduct nutritional assessments for all patients admitted to the ICU. | 4.47 (0.80) |
| **A5** | I believe that the assessment of the nutritional status of ICU patients requires the joint participation of medical staff and nurses. | 4.49 (0.79) |
| **A6** | I believe that choosing the appropriate enteral nutrition formulation/recipe is of great significance in preventing the occurrence of enteral nutrition interruption. | 4.47 (0.79) |
| **A7** | I think the establishment and selection of the enteral nutrition infusion route are very important. | 4.46 (0.80) |
| **A8** | I believe that the management of the feeding position for enteral nutrition is of great significance. | 4.47 (0.80) |
| **A9** | I believe it is extremely important to establish a standardized enteral nutrition management plan to prevent and manage feeding interruptions. | 4.47 (0.80) |
| **A10** | I believe that the prevention of ENI is more important than its treatment. | 4.44 (0.83) |

**Supplementary Table S3. Item-level descriptive statistics for ENI-related practice scores**

| **Practice—Item** | | **Mean (SD)** |
| --- | --- | --- |
| **P1** | I will proactively study the relevant knowledge about the interruption of enteral nutrition. | 4.04 (0.93) |
| **P2** | I will proactively communicate with patients or their families about the importance of enteral nutrition and inform them of the risks associated with the interruption of enteral nutrition. | 4.02 (0.99) |
| **P3** | When admitting patients to the ICU, I will promptly assess their nutritional status and communicate with the attending physician. | 4.21 (0.91) |
| **P4** | Before initiating enteral nutrition, I will communicate with the doctor to select and establish the correct route for enteral nutrition feeding. | 4.26 (0.90) |
| **P5** | During the period of administering enteral nutrition, I will strictly follow the hand hygiene procedures. | 4.49 (0.77) |
| **P6** | In the absence of any medical contraindications, I will raise the head of the bed by 30° to 45° for patients receiving enteral nutrition support. | 4.54 (0.75) |
| **P7** | During the period of enteral nutrition feeding, I will monitor the patient's symptoms of feeding intolerance (such as nausea, vomiting, reflux/aspiration, abdominal distension, etc.), and promptly report them to the physician. | 4.55 (0.73) |
| **P8** | When a patient undergoes emergency establishment or replacement of an artificial airway, such as tracheal intubation or tracheotomy, I will cease enteral nutrition. | 4.52 (0.76) |
| **P9** | When the patient undergoes bedside X-ray imaging and endotracheal bronchoscopy, I will cease the enteral nutrition. | 4.44 (0.88) |
| **P10** | When the patient's condition deteriorates and immediate surgery is required, or when a general anesthesia surgery is scheduled to be performed within 4 hours to 8 hours, I will stop the enteral nutrition. | 4.54 (0.77) |
| **P11** | For patients with feeding intolerance, I will investigate and analyze the causes, and discuss with the physician whether to stop enteral nutrition. | 4.48 (0.79) |
| **P12** | For patients whose shock cannot be corrected, and whose hemodynamic and tissue perfusion targets (MAP < 50 mmHg) cannot be achieved, and who require a gradual increase in the dosage of vasoactive drugs to maintain stable hemodynamics, I will discontinue enteral nutrition. | 4.42 (0.83) |
| **P13** | For patients who develop uncontrollable hypoxemia, hypercapnia or acidosis that poses a threat to their lives, I will cease enteral nutrition. | 4.38 (0.85) |
| **P14** | For critically ill patients with active upper gastrointestinal bleeding or intestinal ischemia, I will stop enteral nutrition. | 4.53 (0.75) |
| **P15** | For patients with elevated intra-abdominal pressure (IAP > 20 mmHg), I will stop enteral nutrition. | 4.43 (0.83) |
| **P16** | During enteral nutrition support, if the gastric residual volume is greater than 250 mL for two consecutive times, I will remind the physician whether to use gastrointestinal motility enhancers. | 4.47 (0.77) |
| **P17** | For patients who have difficulty with enteral feeding and for whom prokinetic drugs are ineffective, as well as those who are considered to have a high risk of aspiration, I will communicate with the physician to establish a post-pyloric feeding route. | 4.45 (0.80) |
