# Supplementary material for: Knowledge, attitudes, and practices regarding enteral nutrition interruption management in intensive care units: a multicenter cross-sectional study predominantly among nurses
Source: Front Nutr. 2026 Oct 2;13:1888995. doi: 10.3389/fnut.2026.1888995 (PMC13671998; doi:10.3389/fnut.2026.1888995)
Supplement: Supplementary file 4 [file Table_2.docx]

**Supplementary Table S4.** Regression diagnostics and sensitivity analyses for the knowledge, attitude, and practice models

| **Model** | **Full-sample n** | **Durbin–Watson** | **Maximum Cook’s D** | **No. with \|standardized residual\| >3** | **Sensitivity n** | **Adjusted R²: full/sensitivity** | **Interpretation** |
| --- | --- | --- | --- | --- | --- | --- | --- |
| Knowledge | 478 | 1.821 | 0.142 | 3 | 475 | 0.141/0.161 | Directions and significance unchanged |
| Attitude | 478 | 1.806 | 0.137 | 3 | 475 | 0.054/0.059 | EN-related training remained significant; sex lost significance, while the tertiary Grade B hospital contrast became significant |
| Practice | 478 | 1.693 | 0.080 | 2 | 476 | 0.126/0.120 | Directions and significance unchanged |

Note. Sensitivity analyses were performed by temporarily excluding observations with absolute standardized residuals >3 and refitting the same multivariable models. Cook’s D = Cook’s distance; R² = coefficient of determination.
