# Supplementary material for: Knowledge, attitudes, and practices regarding enteral nutrition interruption management in intensive care units: a multicenter cross-sectional study predominantly among nurses
Source: Front Nutr. 2026 Oct 2;13:1888995. doi: 10.3389/fnut.2026.1888995 (PMC13671998; doi:10.3389/fnut.2026.1888995)
Supplement: Supplementary file 5 [file Table_3.docx]

**Supplementary Table S5.** Unstandardized and standardized coefficients for all prespecified paths in the observed-variable path model

| **Outcome** | **Predictor** | **B** | **SE** | **z** | ***p*** | **β** |
| --- | --- | --- | --- | --- | --- | --- |
| **Knowledge score** | Tertiary Grade B hospital vs. tertiary Grade A hospital | 1.234 | 1.245 | 0.991 | 0.321 | 0.044 |
|  | Tertiary hospital vs. tertiary Grade A hospital | −1.198 | 3.141 | −0.381 | 0.703 | −0.016 |
|  | Secondary Grade A hospital vs. tertiary Grade A hospital | 1.561 | 1.243 | 1.256 | 0.209 | 0.056 |
|  | Other hospital type vs. tertiary Grade A hospital | −11.464 | 5.367 | −2.136 | 0.033 | −0.091 |
|  | EN team membership, yes vs. no | 2.923 | 1.182 | 2.473 | 0.013 | 0.111 |
|  | EN-related training, yes vs. no | 9.138 | 1.265 | 7.225 | <0.001 | 0.319 |
| **Attitude score** | Knowledge score | 0.456 | 0.024 | 19.167 | <0.001 | 0.690 |
|  | EN-related training, yes vs. no | −0.814 | 0.683 | −1.191 | 0.234 | −0.043 |
|  | Female vs. male | 1.158 | 0.564 | 2.054 | 0.040 | 0.069 |
| **Practice score** | Attitude score | 0.762 | 0.060 | 12.800 | <0.001 | 0.494 |
|  | Knowledge score | 0.302 | 0.042 | 7.246 | <0.001 | 0.296 |
|  | Age 26–30 years vs. ≤25 years | 1.483 | 0.986 | 1.505 | 0.132 | 0.060 |
|  | Age 31–35 years vs. ≤25 years | 0.122 | 1.116 | 0.109 | 0.913 | 0.004 |
|  | Age 36–40 years vs. ≤25 years | 0.673 | 1.111 | 0.606 | 0.545 | 0.023 |
|  | Age ≥41 years vs. ≤25 years | 0.617 | 1.430 | 0.431 | 0.666 | 0.014 |
|  | Tertiary Grade B hospital vs. tertiary Grade A hospital | 2.914 | 0.879 | 3.314 | 0.001 | 0.102 |
|  | Tertiary hospital vs. tertiary Grade A hospital | 4.495 | 2.188 | 2.054 | 0.040 | 0.060 |
|  | Secondary Grade A hospital vs. tertiary Grade A hospital | 2.512 | 0.879 | 2.859 | 0.004 | 0.089 |
|  | Other hospital type vs. tertiary Grade A hospital | −12.253 | 3.763 | −3.257 | 0.001 | −0.095 |
|  | EN team membership, yes vs. no | 1.793 | 0.823 | 2.178 | 0.029 | 0.067 |
|  | EN-related training, yes vs. no | 0.354 | 0.926 | 0.382 | 0.702 | 0.012 |

**Note.** B, unstandardized coefficient; SE, standard error; z, z statistic; β, standardized coefficient. Reference categories were age ≤25 years, tertiary Grade A hospital, no EN team membership, no EN-related training, and male sex. All specified paths, including statistically nonsignificant paths, are presented.
