# Supplementary material for: Knowledge, attitudes, and practices regarding enteral nutrition interruption management in intensive care units: a multicenter cross-sectional study predominantly among nurses
Source: Front Nutr. 2026 Oct 2;13:1888995. doi: 10.3389/fnut.2026.1888995 (PMC13671998; doi:10.3389/fnut.2026.1888995)
Supplement: Supplementary file 6 [file Table_4.docx]

**Supplementary** **Table S6. Nonsignificant factors in the multiple linear regression models**

| **Model** | **Associated factor** | **β** | **SE** | **t** | **p** | **VIF** | **Adjusted R²** | **F** | **Model p** |
| --- | --- | --- | --- | --- | --- | --- | --- | --- | --- |
| **Knowledge** | Hospital type: tertiary Grade B vs. tertiary Grade A hospital | 0.046 | 1.263 | 1.022 | 0.307 | 1.11 | 0.141 | 14.088 | <0.001 |
|  | Hospital type: tertiary hospital vs. tertiary Grade A hospital | −0.014 | 3.189 | −0.316 | 0.752 | 1.04 |  |  |  |
|  | Hospital type: secondary Grade A vs. tertiary Grade A hospital | 0.058 | 1.262 | 1.281 | 0.201 | 1.14 |  |  |  |
| **Attitude** | EN team membership (yes vs. no) | 0.053 | 0.834 | 1.118 | 0.264 | 1.14 | 0.054 | 4.893 | <0.001 |
|  | Hospital type: tertiary Grade B vs. tertiary Grade A hospital | 0.081 | 0.880 | 1.716 | 0.087 | 1.12 |  |  |  |
|  | Hospital type: tertiary hospital vs. tertiary Grade A hospital | −0.031 | 2.211 | −0.685 | 0.494 | 1.04 |  |  |  |
|  | Hospital type: secondary Grade A vs. tertiary Grade A hospital | −0.027 | 0.875 | −0.564 | 0.573 | 1.14 |  |  |  |
|  | Hospital type: other vs. tertiary Grade A hospital | −0.081 | 3.785 | −1.792 | 0.074 | 1.03 |  |  |  |
| **Practice** | Age group: 31–35 years vs. ≤25 years | 0.068 | 1.687 | 1.206 | 0.228 | 1.73 | 0.126 | 7.898 | <0.001 |
|  | Age group: 36–40 years vs. ≤25 years | 0.024 | 1.680 | 0.424 | 0.671 | 1.74 |  |  |  |
|  | Age group: ≥41 years vs. ≤25 years | 0.074 | 2.161 | 1.477 | 0.140 | 1.38 |  |  |  |
|  | Hospital type: tertiary hospital vs. tertiary Grade A hospital | 0.041 | 3.308 | 0.930 | 0.353 | 1.05 |  |  |  |
|  | Hospital type: secondary Grade A vs. tertiary Grade A hospital | 0.081 | 1.326 | 1.739 | 0.083 | 1.20 |  |  |  |
